# The Dynactin Complex Enhances the Speed of Microtubule-Dependent Motions of Adenovirus Both Towards and Away from the Nucleus

**DOI:** 10.3390/v3030233

**Published:** 2011-03-09

**Authors:** Martin F. Engelke, Christoph J. Burckhardt, Matthias K. Morf, Urs F. Greber

**Affiliations:** 1 Institute of Molecular Life Sciences, University of Zurich, Zurich 8057, Switzerland; E-Mails: martin.engelke@imls.uzh.ch (M.F.E.); christoph_burckhardt@hms.harvard.edu (C.J.B.); matthias.morf@imls.uzh.ch (M.K.M.); 2 Molecular Life Sciences Graduate School, ETH and University of Zurich, Zurich 8057, Switzerland

**Keywords:** dynein, motor, cytoplasmic transport, cytoskeleton, virus motion, infection

## Abstract

Unlike transport vesicles or organelles, human adenovirus (HAdV) directly binds to the microtubule minus end-directed motor dynein for transport to the nucleus. The dynein cofactor dynactin enhances nuclear transport of HAdV and boosts infection. To determine if dynactin has a specific role in cytoplasmic trafficking of incoming HAdV on microtubules, we used live cell spinning disc confocal microscopy at 25 Hz acquisition frequency and automated tracking of single virus particles at 20–50 nm spatial resolution. Computational dissection by machine-learning algorithms extracted specific motion patterns of viral trajectories. We found that unperturbed cells supported two kinds of microtubule-dependent motions, directed motions (DM) and fast drifts (FD). DM had speeds of 0.2 to 2 μm/s and run lengths of 0.4 up to 7 μm, while FD were slower and less extensive at 0.02 to 0.4 μm/s and 0.05 to 2.5 μm. Dynactin interference by overexpression of p50/dynamitin or a coiled-coil domain of p150/Glued reduced the speeds and amounts of both center- and periphery-directed DM but not FD, and inhibited infection. These results indicate that dynactin enhances adenovirus infection by increasing the speed and efficiency of dynein-mediated virus motion to the nucleus, and, surprisingly, also supports a hereto unknown motor activity for virus transport to the cell periphery.

## 1. Introduction

Viruses receive extensive assistance from the host cells for infection. To enter into cells, they utilize receptors for attachment and movement on the cell surface [1], or employ signaling machineries and endocytic pathways; for reviews, see [2–4]. They escape to the cytosol by membrane fusion in the case of enveloped viruses [5], or membrane disruption in the case of nonenveloped viruses [6]. In the cytosol, subviral particles consisting of protein-encapsidated genomes are trafficked to sites for replication, and this trafficking often involves microtubules [7–9]. Many viruses that replicate in the nucleus use the dynein/dynactin motor complex to traffic to the nucleus [10,11]. Cytoplasmic dynein is responsible for a broad range of cellular functions, including transport of organelles and regulation of mitosis [12–14]. It is a large protein complex composed of different subunits, and moves in the range of μm/s towards microtubule minus ends, which are organized in the centrosome near the nucleus of nonpolarized or migratory cells [15]. In polarized cells, however, microtubule minus ends are oriented away from the nucleus. The usage of multiple motors hence might be particularly advantageous for viruses, which utilize the nucleus for genome replication. Such viruses comprise influenza viruses, herpes viruses or small DNA-tumor viruses, such as human and animal polyomaviruses, papillomaviruses and human adenoviruses (HAdV).

Currently, there are 56 HAdV types known, which are grouped into seven species A to G [16]. HAdV are transmitted by respiratory, fecal-oral or fomital routes, and cause respiratory, ocular, genito-urinary and gastrointestinal disease. They are nonenveloped viruses and encapsidate a double-stranded DNA genome of about 36 kbp. HAdVs were the first respiratory disease causing viruses isolated and characterized [17,18]. Adenoviruses also have a longstanding history and have been an amazing discovery tool for molecular cell biology. Key host cell functions that have been discovered with HAdV include RNA splicing [19,20], interference with class 1 major histocompatibility antigen presentation [21], chaperone-mediated protein folding with the viral hexon protein and the 100K folding factor [22], DNA replication [23], and paradigms for DNA-tumor viruses, particularly HAdV12 in animals [24,25]. More recently, extensive efforts have been made to develop adenoviruses into gene delivery vehicles for therapy of diseased cells, or eradication of tumor cells; for a review see [26].

The adenovirus particle is composed of 12 viral proteins encoded by distinct open reading frames. Most of these proteins build up the capsid, and some are associated with the viral DNA enclosed within the capsid. The icosahedral capsid of the species C HAdV2 or its quasi-equivalent HAdV5 has a triangulation number T = 25, and is composed of the major protein hexon and a number of cementing proteins, which secure the stability of the virion [27–29]. Four viral proteins are known to be located on the outside of the virion, hexon, protein IX (pIX), penton base and fiber. Fiber proteins protrude from the 12 vertices and attach the virus to the cell adhesion receptor CAR (coxsackievirus adenovirus receptor), see [30,31]. Alpha v beta 3/5 integrins facilitate dynamin-dependent endocytosis by binding to penton base, which anchors the fibers to the particle [32–34]. Viruses then escape from an early endosome to the cytosol by activating the internal protein VI [35,36]. Endosomal escape is coupled to a stepwise viral uncoating program during entry. Upon engagement with CAR and integrins, the virions shed fibers, and release the internal cementing proteins IIIa, V, VI and VIII [35,37,38].

A membrane-free subviral particle is released into the cytoplasm. It consists of hexon, a fraction of penton base and pIX, which secures the facets and organizes the group of nine hexons, and the viral DNA condensed with pV and pVII, and possibly the hexon-associated pVI. The particle then translocates towards the nucleus in a bidirectional transport mode that depends on microtubules and dynactin [39–43]. Stochastic *in silico* simulations matching the microtubule-dependent motions of wild type HAdV2 or pIX-deleted HAdV2 suggested that during active translocations, 1–3 motors of either nuclear or peripheral polarity were engaged simultaneously with one virus particle, although up to 15 binding sites per virion were estimated to be available for binding [44]. Biochemical binding studies with isolated virions and subviral particles then showed that the dynein subunit IC74 (intermediate chain 74 kDa) and LIC1 (light intermediate chain 1) interacted with hexon [45]. Functional significance for these interactions was furnished by microinjections of anti-dynein or anti-hexon antibodies, which inhibited the translocation of cytosolic particles to the nucleus and infection [45,46]. Other viral proteins, such as pVI or protease, have also been proposed to bind dynein or have suggested to be involved in virus transport in the cytoplasm, but their role is not firmly established [47,48]. After reaching the nuclear envelope, the subviral particle attaches to the nuclear pore complex by binding to Nup214/CAN, and releases its DNA into the nucleus [49–51].

The mechanisms by which the subviral particles reach the nuclear pore complex are still incompletely understood. Here, we aimed to clarify the role for the dynein cofactor dynactin in microtubule-dependent transport of particles towards the nucleus. Dynactin was initially found as a cytosolic factor that activates dynein and enhances dynein processivity [52,53]. It has been implicated in many additional functions, such as cargo binding and loading onto microtubules or coordinating the activities of opposing motors; for reviews, see [54–56]. Dynactin comprises a rod like filament mainly composed of Arp1 (actin-related protein 1). This filament is tethered to p150/Glued by four p50/dynamitin subunits. p150/Glued forms an extended antenna-like structure, which binds to microtubules by a CAP-Gly domain, and is thought to contact not only cytoplasmic dynein but also one or several kinesins. Overexpression of the dynactin subunit p50/dynamitin or the first α-helical coiled-coil domain (CC1) of p150/Glued disrupts the intra- and inter-subunit contacts of dynactin, and thereby inhibits a wide variety of microtubule-dependent transport processes of cellular and viral cargoes [8,56,57]. Here we specifically show that the overexpression of p50/dynamitin or CC1 of p150/Glued blocks the fast bidirectional trajectory segments of HAdV with run lengths in the range of 0.4–7 μm and thereby inhibits infection.

## 2. Results

### 2.1. Intact Microtubules are Required for Adenovirus Infection and Support Two Types of Directional Movements of Incoming Virus Particles

**Figure 1.**
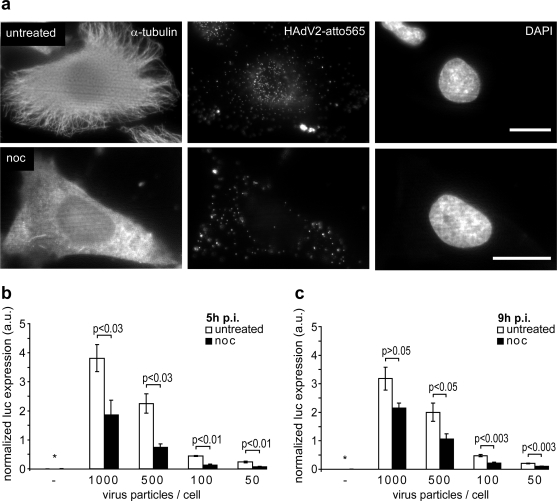
Adenovirus infection requires intact microtubules. (**a**) HeLa cells were kept in the presence or absence of nocodazole for 30 min, inoculated with 0.5 μg HAdV2-atto565 and incubated at 4 °C for 30 min. Temperature was shifted to 37 °C and infection proceeded for 90 min, cells were fixed with PHEMO fixative [42], processed for DAPI and immunostaining against alpha-tubulin and imaged by wide field fluorescence microscopy. Scale bars 20 μm. (**b**, **c**) HeLa cells were continuously treated or left untreated with nocodazole in Hoechst containing medium, infected with the indicated physical virus particle to cell ratios for 5 h (**b**) or 9 h (**c**), lysed and luciferase expression was quantified. Before cell lyses and in the last 15 min of infection, cells were imaged with an ImageXpress Micro Imaging Station (Molecular Devices) in the Hoechst channel. Luciferase expression was normalized to the number of nuclei detected in the 16 sites imaged per well. An asterisk indicates samples with luciferase expression below the detection limit. The means ± SEM of 4 (**b**) or 3 (**c**) independent experiments are shown. The two-tailed Student's t test was used to determine p-values.

We first tested if HAdV5 infection of HeLa cells was microtubule-dependent. Cells were either treated or not treated with 40 μM nocodazole to depolymerize microtubules, and inoculated with HAdV5 encoding luciferase. Nocodazole depleted the microtubules as indicated by anti-α-tubulin staining (Figure 1a). It reduced luciferase expression at different multiplicities of infections (MOI) both 5 and 9 h post infection (Figure 1b, c). These data are in agreement with earlier results from human bronchial epithelial A549 or African green monkey kidney cells, where microtubules enhanced HAdV5 infection without affecting the efficiency of viral escape from endosomes [39,42].

**Figure 2.**
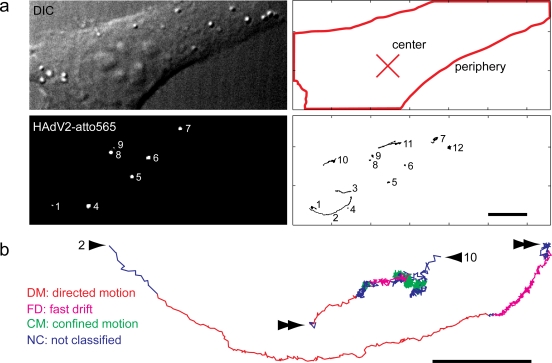

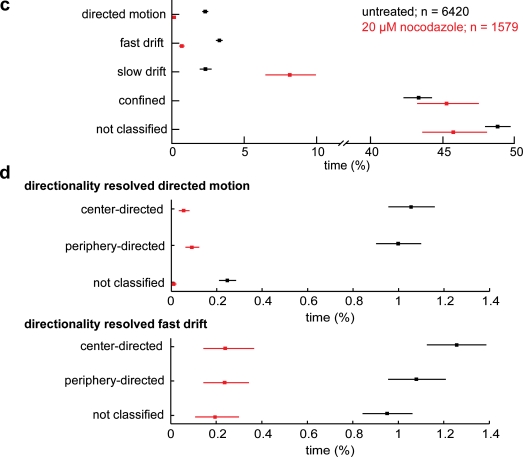
Live cell imaging and trajectory segmentations reveal distinct HAdV motion patterns. (**a**) HeLa cells were infected with HAdV2-atto565, and imaged between 30–90 min p.i. Time stacks were processed with a particle tracker yielding single virus trajectories. Trajectories of viruses that are visible for at least 100 frames are show. The size and intensity of the signal recorded from the virus particles depends on the imaging plane, and the particles brightness. Viruses and corresponding trajectories are numbered (1–12). Please note that virus 1 gives rise to trajectories 1–3, for example, since this virus is moving in and out of the imaging plane (see also Supplemental Movie 1). Trajectories 10–12 are from viruses that are not visible in the particular still image, but are visible in the supplemental movie. Scale bar 8 μm, cross indicates the center of the nucleus. (**b**) Directed motions (DM, red), fast drifts (FD, pink), confined motions (CM, green) and not classified (NC, blue) as identified machine learning and trajectory segmentation. Arrow head indicates the start, double arrow heads the end of two trajectories, and numbers correspond to particles and trajectories in panel (a). Scale bar 2 μm. (**c**) The number of motion steps of a particular motility type is shown as a fraction of the total number of motion steps. The median and the 95% confidence interval obtained by bootstrapping are shown; n = number of tracks. (**d**) The directionality of the motion segments from the data set in (c) was classified as described in the Materials and Method section with the median and the 95% confidence interval obtained by bootstrapping.

We next analyzed the motion patterns of cytosolic HAdV2 particles. For this, we performed live cell, spinning disc confocal microscopy to observe the motions of single virus particles at high spatio-temporal resolution (Figure 2a, and Supplemental Movie 1). HAdV2 particles labeled with the organic dye Atto565 (HAdV2-atto565) were bound to untreated or nocodazole treated cells in the cold. Movies with a length of 80 s (equivalent to 2000 frames at 25 Hz acquisition frequency) were acquired between 30 and 90 min post warming at 37 °C. At these time points the majority of virus particles are in the cytosol and only 10 to 20% are in endosomes as indicated by transmission electron microscopy analyses [58]. Fluorophore-tagged viruses in endosomes at 30 min or later post infection (p.i.) can be readily distinguished from cytosolic particles by their heterogeneous intensities due to accumulation of multiple particles in individual late endosomes or lysosomes [34,39]. For analyses of individual cytosolic particles we used a particle tracker with a precision of approximately 20–50 nm resolution [59], and analyzed particle trajectories from the homogenous population using a previously published trajectory segmentation algorithm based on specific training data and support vector machines [60].

In control cells we found so-called directed motion (DM) segments, which were composed of unidirectional steps and had rather high overall speeds of 0.2 to 2 μm/s and long run lengths of 0.4 up to 7 μm (Figure 2b, Figure 5c). DM accounted for about 2% of the live time of the particles in the cytoplasm (Figure 2c). We also detected unipolar fast drifts (FD), which were slower (0.02 to 0.4 μm/s) and shorter (0.05–2.5 μm) than DM. Their abundance was similar to the DM. In addition to DM and FD, we found slow drifts (SD) [60], which resembled FD in terms of abundance but had lower speed (0.002 to 0.015 μm/s). Finally, we observed extensive periods of confinement in areas of about 100 nm in diameter (data not shown), and [60].

These confinements had no detectable overall movement directionality and accounted for more than 40% of the live time of the particles in the cytoplasm. All the motion steps that did not fall into any of the above described categories were assigned as not classified. They comprised nearly 50% of the particles life time, and remain to be mechanistically explored in the future. Importantly, in nocodazole-treated cells, the abundance of DM and FD were significantly reduced compared to control cells, while all other motion patterns were similar or more abundant than in control cells (Figure 2c).

**Figure 3.**
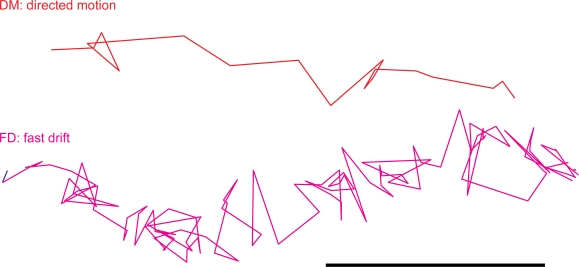
Representative examples for directed motions (DM) and fast drifts (FD). HeLa cells infected with HAdV2-atto565 were imaged and a particle tracker was used to obtain single virus trajectories. Trajectories were segmented and short stretches of a directed motion (DM, red) and a fast drift (FD, magenta) are shown. Scale bar represents 160 nm.

We next assigned a directionality feature to DM and FD, namely center or periphery direction. Motion segments running perpendicular to the axis between the particle and the center of the nucleus were termed ‘non-classified’. Nocodazole strongly reduced both the periphery and center-directed DM and FD from about 1% to 0.1 to 0.2% in each case, and also significantly depleted the non-classified DM and FD (Figure 2d). We conclude that both DM and FD are microtubule-dependent motility types of incoming HAdV2 and are directed towards or away from the nucleus (for enlarged examples of DM and FD, see Figure 3).

### 2.2. Dynactin Enhances Infection and Supports Long and Fast Center- and Periphery-Directed Adenovirus Motion Runs

**Figure 4.**
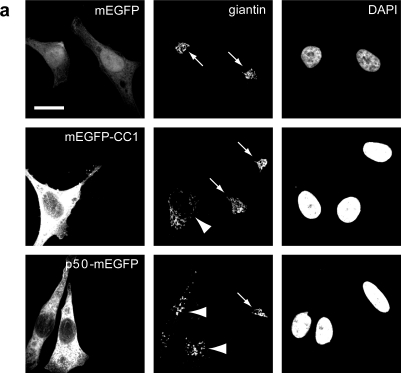

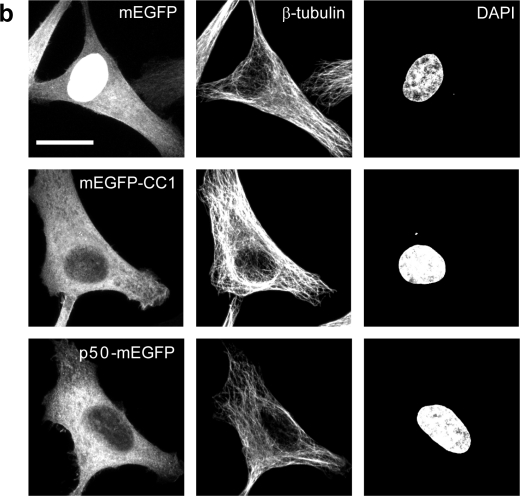
Overexpression of p50-mEGFP or mEGFP-CC1 induces Golgi fragmentation. HeLa cells were seeded on glass cover slips and transiently transfected with mEGFP, p50-mEGFP, or mEGFP-CC1 for 16 h, fixed with (**a**) paraformaldehyde or (**b**) PHEMO fix and processed for DAPI and immunostaining against (**a**) giantin or (**b**) β-tubulin. Scale bars 20 μm.

Recent biochemical evidence suggested that dynein is recruited to HAdV5 by direct interaction of the intermediate chain 74 and light intermediate chain 1 with the hexon protein of the capsid [45]. Based on p50/dynamitin overexpression [61], it was also noticed that dynactin enhanced HAdV2/5 infection [39,45]. To address the question of how dynactin is involved in HAdV infection, we overexpressed two peptides of the dynactin complex, p50/dynamitin tagged to monomeric A206K-EGFP (here referred to as mEGFP) and CC1 domain from p150/Glued tagged to mEGFP. mEGFP has minimal hydrophobic aggregation and self-association, and hence is well suited for mechanistic analyses [62]. Overexpression of p50-mEGFP or mEGFP-CC1 induced scattering of the Golgi apparatus 16 h post transfection as judged by indirect immunofluorescence staining of giantin (Figure 4a).

**Figure 5.**
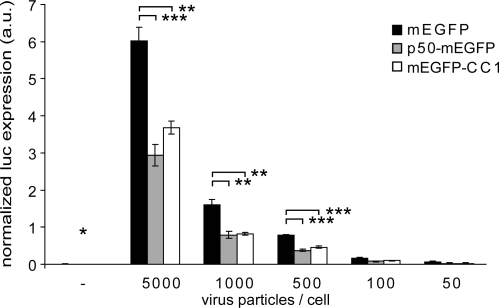
Dynactin interference inhibits HAdV5 transduction. HeLa cells were transiently transfected with mEGFP, p50-mEGFP or mEGFP-CC1 for 16 h, incubated in Hoechst containing medium and infected with the indicated virus particle to cell ratios for 9 h. Cells were imaged with an ImageXpress Micro Imaging Station (Molecular Devices) in the Hoechst and mEGFP channel 30 min before lysis and luciferase expression was detected, normalized to the average mEGFP expression and to number of nuclei detected in 16 sites imaged per well. The mean values ± SEM of three independent experiments are shown. * indicates samples with luciferase expression below the detection limit, ** p < 0.02, *** p < 0.005.

**Figure 6.**
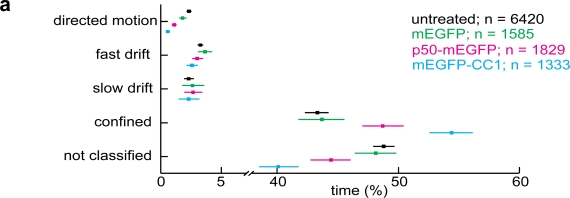

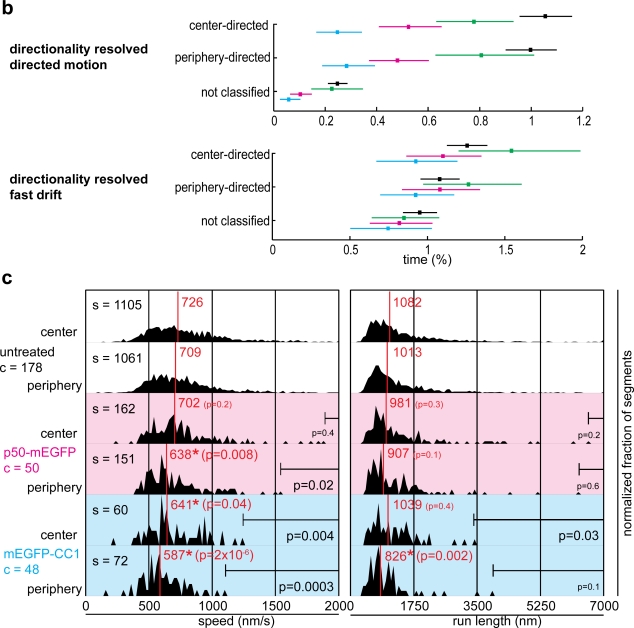
Dynactin supports long and fast microtubule-dependent motion runs. HeLa cells were seeded on glass cover slips and left untransfected or transiently transfected with mEGFP, p50-mEGFP or mEGFP-CC1 for 16 h. Cells were infected with HAdV2-atto565 and viral trajectories acquired 30–90 min p.i. with number of tracks (n). (**a**) The time that the viruses spent in certain motility types is shown as a fraction of total time with the median and the 95% confidence interval obtained by bootstrapping. (**b**) The data shown in (a) were grouped according to their directionality with the median and 95% confidence interval. (**c**) Representation of speed and run length histograms for DM runs. The frequency was normalized to the number of total DM for each condition. Note that the dataset for untreated cells is considerably larger than the datasets for the other conditions, and therefore the histogram for untreated cells appears smoother. Vertical red lines and red numbers with p-values in parentheses indicate the median for each dataset. Asterisks indicate medians that are significantly lower (*) than the median of untreated cells (p < 0.05). A threshold zone is indicated by a horizontal black line with the p-value representing the probability to draw a random sample (which has the size s as indicated) from the untreated dataset, which does not contain any value above the indicated threshold. c = number of cells; n = number of tracks; s = number of segments.

As expected, the overexpression of p50-mEGFP or mEGFP-CC1 did not apparently alter the β-tubulin staining in PHEMO-fixed cells, as indicated by indirect immunofluorescence microscopy (Figure 4b), in agreement with earlier observations [61]. It did, however, inhibit HAdV5 infection, as indicated by luciferase transgene expression at MOI 50 to 5000 physical particles/cell (corresponding to 2 to 200 infectious particles/cell) compared to cells expressing mEGFP alone (Figure 5). We concluded that dynactin is required for efficient HAdV2/5 infection. Motility analyses showed that the expression of p50-mEGFP or mEGFP-CC1 inhibited the DM but not FD or SD, and enhanced the confined motions (Figure 6a). Directionality analyses indicated that, surprisingly, both the center- and the periphery-directed DM were reduced by the expression of p50-mEGFP or mEGFP-CC1 (Figure 6b).

To further examine the effects of dynactin inhibition on adenovirus motions, we extracted single DM run properties, such as run length and speed. The results show that mEGFP-CC1 significantly reduced the median speeds of center- and periphery-directed DM runs from 726 to 641 nm/s, and 709 nm/s to 587 nm/s, respectively, compared to untreated cells (Figure 6c). More specifically, mEGFP-CC1 inhibited the center-directed runs faster than 1243 nm/s (p = 0.004), and the periphery-directed runs faster than 1105 nm/s (p = 0.0003). It also reduced the median run length of periphery-directed runs from 1013 nm to 826 nm, and specifically the run lengths of center-directed runs longer than 3407 nm (p = 0.03). p50-mEGFP expression reduced the median speed of periphery-directed runs from 709 nm/s to 638 nm/s, and specifically inhibited periphery-directed runs faster than 1541 nm/s (p = 0.02). The distribution of DM run lengths and speeds from mEGFP transfected cells were similar to untreated cells also containing high values for run length and speeds (data not shown). Taken together, these results support a key role of dynactin in fast and long motions of HAdV2/5 either directed to the periphery or the center. The data imply that besides the minus end-directed microtubule motor dynein and its cofactor dynactin, a plus end-directed motor could be important for the fast movement of adenovirus particles in the cytosol, and could also enhance infection, similar to dynein. Furthermore, the number of DM runs per track was reduced from 31% in untreated cells to 11% and 19% respectively, in mEGFP-CC1 and p50-mEGFP expressing cells (Table 1).

## 3. Discussion

The typical motion pattern of cellular and viral cargoes in the cytoplasm resembles stop-and-go traffic in forward and backward directions, and rarely looks like smooth gliding to the destination of interest. It is thought that bidirectional movements on microtubules help the attached motors to navigate cargo through a crowded cytoplasm. Rapid switching between plus and minus end-directed movements may occur stochastically, or aspects thereof may be regulated, for example, at the level of the cargo, the motor or the microtubule tract [63,64].

It is possible, though unlikely, that at any given time point there is only one motor-type bound to the cargo, and another motor-type is recruited to switch the transport directionality. Perhaps more likely, both types of motors are simultaneously bound to the cargo, but only one type is active during directional motion runs, or one type of motor is more abundant on the cargo than the other. Switching directionality may occur by yet unknown regulatory mechanisms, or may occur stochastically in an unregulated tug-of-war between motors of opposite directionality simultaneously bound to the cargo [44,65].

For adenovirus, the on and off-rates of motors from microtubules (or cargo) and the motor displacement rates have been modeled by stochastic simulation and shown to match the viral movements on microtubules in live cells [44]. This suggested that directional movements of adenovirus can arise by stochastic binding, unbinding and stepping of motors, and bidirectionality may be a consequence of resolving tug-of-war situations.

Besides stochasticity, there are aspects of motor function in adenovirus infection that may be regulated. For example, incoming HAdV2/5 activate a signaling cascade involving protein kinase A and p38/MAPK, which favor dynein/dynactin-mediated translocation of subviral particles towards the nucleus [41]. Dynactin is an important cofactor for dynein and enhances adenovirus infection [39,43].

Using wide field fluorescence microscopy at low temporal resolution of 1 Hz and focusing on trajectories larger than 100 motion steps, these studies found that the overexpression of p50/dynamitin reduced the mean speed of both center- and periphery-directed motion steps and also the number of steps directed to the nucleus, and increased the motion-free periods.

Since p50 can bind proteins other than dynactin, such as MacMARCKS or BICD2 [66,67], we extended these studies by expressing the first coiled-coil domain CC1 of the dynactin subunit p150/Glued. CC1 expression saturates the dynactin binding site on dynein, and thereby displaces dynactin from dynein motor. Virus movement analyses were conducted at high temporal resolution of 25 Hz, which is a substantial improvement in temporal resolution compared to earlier studies [39]. This allowed us to use automated virus tracking with high fidelity.

Our new results show that interference with mEGFP-CC1 or p50-mEGFP suppressed the fast microtubule-dependent speeds of cytosolic HAdV2. Remarkably, mEGFP-CC1 expression significantly reduced both center- and periphery-directed motions, while p50-mEGFP significantly reduced the periphery-directed speeds and slightly the center-directed speeds. This suggests that dynactin directly or indirectly regulates dynein and a plus end-directed motor transporting HAdV2/5. This reflects earlier observations in *Drosophila* neuronal cells, where interference with normal dynactin assembly by genetic mutations or RNA interference disrupted both anterograde and retrograde transport of organelles, but did not interfere with dynein attachment to cargo [68].

Therefore, certain aspects of opposing motor functionality are likely to be coordinated by dynactin. Recent evidence from cell cultures supports this notion. It was shown that microtubule-dependent transport of peroxisomes in center or periphery direction required two opposite polarity motors, dynein and kinesin-1 [69]. These organelles stalled, if either kinesin-1 or dynein was absent. Peroxisome movement in both directions could be restored when a different kinesin was ectopically targeted to the organelle, suggesting that dynein-mediated runs require a plus end-directed opposing motor.

Concerning adenovirus transport, we suggest that dynein and an unknown plus end-directed motor are coordinated by dynactin to enhance the bidirectional movements of HAdV2/5. By providing a mechanical link between motors of opposite polarity, dynactin could activate one of the opposing motors, and enhance the probability that one of the motors starts running. Biochemical work in melanophores showed that dynactin can bind to dynein or in this case kinesin-2 but not simultaneously, suggesting a mechanism by which dynein could regulate direction [70]. Once the motor is running, dynactin may balance the opposing motor, and thereby enable the fast DM motions of adenovirus.

Interestingly, a basic sequence motif in p150/Glued is thought to function as a skater and progressively glides along microtubules, thereby increasing the processivity of dynein and prolonging the interaction time of the motor with microtubules [71,72]. The CAP-Gly domain of p150/Glued on the other hand leads to motor stalling but keeps the motor on microtubules [71].

Interference with dynactin suppressed the processive DM but not the FD runs of adenovirus. This suggests that FD are mediated by dynein and the opposing motor alone without functional dynactin. This scenario is similar to a proposed role for dynactin in kinesin-2 and dynein-mediated bidirectional movement of melanosomes in *Xenopus laevis* melanophores, where p150/Glued recruits the cargo binding portion of kinesin-2 [70], and enhances the processivity of kinesin-2 in cell-free systems [73]. This model is also consistent with the notion that dynein binds directly to the viral hexon [45]. To establish the biophysical difference between DM and FD in molecular terms, individual motor stepping events need to be resolved by enhanced imaging and further increased spatio-temporal resolution of virus trajectories.

## 4. Materials and Methods

### 4.1. Cells, Viruses and Plasmids

HeLa cells were kindly provided by U. Kutay (ETH Zurich, Switzerland) and human epithelial KB cells were obtained from ATCC (American Type Culture Collection, USA). Cells were subconfluently cultured in Dulbecco’s modified Eagle’s medium (D-MEM) supplemented with 2 mM Glutamine (Sigma-Aldrich, Buchs, Switzerland), 10% fetal bovine serum, and 1% nonessential amino acids (Gibco-BRL, Invitrogen, Basel, Switzerland). HAdV2 was propagated in and isolated from KB cells. Viruses were purified and labeled with atto565 (Atto-tec, Siegen, Germany) as previously described [37]. The luciferase reporter virus Luc-Ad5 is described in [42] and was kindly provided by S. Hemmi (University of Zurich). The EGFP-N1 and EGFP-C1 plasmids were obtained from Clontech (BD Biosciences, USA).

The construct mEGFP-CC1 was cloned by inserting the chicken CC1 domain of p150/Glued [57] into the pEGFP-C1 vector (Clontech) employing the restriction sites EcoRI and BamHI. The restriction sites were introduced into the CC1 domain using the following primers: CGGAAT-TCCGAGGAAGAAAATCTGCGTTCC and CGGGATCCCGCTACTGCTGCTGCTTCTCTGC. The construct p50-EGFP was kindly provided by R. Vallee (Columbia University, NY, USA). In both plasmids mentioned above, the A206K mutation [74] was introduced into EGFP employing the following primers: CCTGAGCACCCAGTCCAAGCTGAGCAAAGACCCCA and TGGGGTC-TTTGCTCAGCTTGGACTGGGTGCTCAGG according to the strategy described in the manual for the QuikChange Site-Directed Mutagenesis Kit (Stratagene, Texas, USA).

### 4.2. Luciferase Assay

For dynactin interference experiments, HeLa cells were transfected with the Neon Transfection System (Invitrogen, Basel, Switzerland) according to the manufacturer’s protocol. For all other experiments cells were left untreated. Subsequently, 25,000 cells were seeded into an imaging compatible 96-well imaging plates (Matrix) kept in full D-MEM medium in a humidified incubator for 16 h at 37 °C and 5% CO_2_. The next morning, medium was replaced by HEPES buffered RPMI-1640 medium (Sigma-Aldrich, Buchs, Switzerland) supplemented with 0.2% bovine serum albumin (Sigma-Aldrich, Buchs, Switzerland). For all nocodazole conditions the media throughout the experiment contained 40 μM nocodazole (Sigma-Aldrich, Buchs, Switzerland). Cells were kept at 4 °C for 30 min, and subsequently shifted to 37 °C for 1 h. Medium was replaced by full D-MEM medium containing Hoechst 33342 (Sigma-Aldrich, Buchs, Switzerland) and Luc-Ad5. Infection proceeded for 5 h or 9 h at 37 °C and 5% CO_2_. During the last 30 min of infection, plates were sealed with translucent tape, and cells were imaged in an ImageXpress Micro (Molecular Devices, Ismaning, Germany) automated imaging station. The station is equipped with a CERMAX arc Xenon lamp (PerkinElmer, Schwerzenbach, Switzerland) for bright illumination, an S Fluor 10x objective, as well as BrightLine filter cubes (DAPI-5060B-NTE-ZERO and GFP-3035B-NTE-ZERO, Semrock, Rochester, NY). For each well, 16 sites were imaged in the DAPI and GFP channels. The imaging chamber was heated to 37 °C during image acquisition. Subsequently, cells were washed with PBS, and lysed with reporter lysis buffer (RLB, Promega, Mannheim, Germany) according to the manufacturer’s protocol. Luciferase expression was detected with the Luciferase Assay System and a GloMax Multi Detection System (Promega, Mannheim, Germany). The number of cells per well was determined in DAPI images and the average fluorescent intensity around the nuclear rim was measured from GFP images with custom written MATLAB (MathWorks, Natick, MA, USA) scripts. The luciferase signal was normalized to the number of nuclei detected on 16 images per well and normalized to the mEGFP expression level if applicable.

### 4.3. Transfections and Microscopy of Immunostained Cells

Cells were seeded with 30% confluency on cover glasses (Assistent, Germany) and were transfected 6 h later using FuGENE 6 Transfection Reagent (Roche Diagnostics, Mannheim, Germany) and serum free D-MEM according to the manufactures protocol. On the next day, cells were either fixed with 3% paraformaldehyde (pFA) for 15 min or PHEMO fixative [42] for 10 min, treated with 25 mM ammonium chloride in PBS for 10 min and permeabilized with 0.5% Triton X-100 in PBS for 5 min. Mouse anti-Giantin antibody (H.P. Hauri, University of Basel, Switzerland) or anti-β-tubulin antibody (N357, Amersham) or anti-α-tubulin antibody (1A2) [75] was diluted in PBS, containing 10% goat serum (Gibco-BRL, Invitrogen, Basel, Switzerland) and applied to the samples for 1 h at room temperature. Subsequently, samples were washed with PBS and immunostained with goat anti-mouse-Texas Red (Jackson Immunoresearch, PA, USA) or Alexa Fluo 488 goat anti-mouse (Invitrogen, Basel, Switzerland) diluted in PBS containing 10% goat serum and DAPI (Molecular Probes, Leiden, The Netherlands) with the concentration indicated by the manufacturer for 15 min at room temperature. Cells were washed with PBS and mounted in 4 μL mounting medium (DAKO, Carpinteria, CA, USA) on object slides (Menzel Gläser, Braunschweig, Germany). Images were acquired either with an Olympus IX81 inverted epifluorescence microscope equipped with a PlanApo 60x oil immersion objective controlled by the CellM software (Olympus Optical AG, Switzerland) or with an Leica SP5 scanning confocal microscope controlled by Leica LAS AF software (Leica Microsystems, Wetzlar, Germany).

### 4.4. Live Cell Imaging

For live cell imaging, cells were seeded at 20% confluency on 18 mm cover slips imaging (Menzel Gläser, Braunschweig, Germany) and transfected as described above. On the next day, cells were either treated with 20 μM nocodazole (Sigma-Aldrich, Buchs, Switzerland) in D-MEM or left untreated for 30 min. Subsequently, they were washed with PBS and 0.8 μg of HAdV2-atto565 were applied to the cells. Viruses were allowed to bind to the cell surface at 4 °C in RPMI medium (Gibco-BRL) supplemented with 0.2% BSA for 30 min, washed with serum free D-MEM containing 0.2% BSA and warmed to 37 °C in an incubator containing 5% CO_2_.

Time-lapse fluorescence microscopy was performed 30 to 90 min post warming in Hank’s Buffered Salt Solution (Gibco-BRL) supplemented with 0.5% BSA and 1 mg/mL ascorbate at pH 7.3 in a temperature controlled incubator box at 37 °C (Live Imaging Services, Switzerland). Spinning disc confocal microscopy was conducted with a Yokagawa disc system on an Olympus IX81 inverted microscope equipped with a Yokogawa scanning head QLC100 (VisiTech International, UK) a triple bandpass dichroic mirror (488 nm/565 nm/647 nm, Chroma, USA) and a N.A. 1.35 UPlanApo 100x oil immersion objective (Olympus Optical AG, Switzerland). Images were recorded onto a 512 × 512 pixel chip (16 × 16 μm per pixel) of a cooled, back-illuminated, 16-bit monochrome CCD camera (Cascade 512, Roper Scientific, USA). Fluorophores were excited with an Innova 70C mixed-gas laser (Coherent, Germany) with 488 nm and 568 nm, respectively. The microscope and camera were controlled with the Metamorph software package (Universal Imaging, USA).

### 4.5. Data Analysis

Time stacks comprising 2000 frames taken at 25 Hz were processed with a single particle tracking software [59] to extract 2D trajectories of moving virions. The signal-to-noise ratio was 2.5 allowing to determine the virus particle position with an estimated accuracy of 20–50 nm [59]. Along with each time stack an image of the cell in the DIC (differential interference) channel was taken. Visual inspection of the DIC images allowed the extraction of the center coordinates of the cell nucleus. Further post processing procedures were implemented in MATLAB (MathWorks, Natick, MA, USA). Trajectories of virions that were visible in less than 100 of the 2000 frames were removed and remaining trajectories were processed with a supervised trajectory segmentation algorithm based on Support Vector Machines as classifiers [60]. This procedure aimed at the detection of distinct, non-random, motion patterns in HAdV2 trajectories. Trajectory segments were classified as confined motions (CM), slow drifts (SD), fast drifts (FD), directed motions (DM) and not classified.

For each segment the distance from the segment start point coordinates to the cell center (d S-C) was compared with the distance of the cell center to the segment endpoint coordinates (d C-E). If d S-C > d C-E, the corresponding segment was classified as center-directed. If d S-C < d C-E, the segment was classified as periphery-directed. Segments with a distance difference of d S-C and d C-E less than one image pixel (160 nm) were directionally not classified. These motion segments corresponded to tangential virus movement with respect to the nucleus. The run length of a segment was defined as the net distance from the start point of the segment to its endpoint. Taking the imaging frequency into consideration, the duration of a segment was calculated from the number of frames of the track. Dividing the segment run length by its duration yielded the segment speed. A particle-frame was defined as one particle that is visible during one frame. The abundance of one motility type is calculated as the number of particle-frames that virions spent in a certain motility type divided by the number of particle-frames of all virions.

Populations of DM speeds and run length were characterized by calculating median speeds (nm/s) and run lengths (nm) with the according probabilities that they are different from the dataset of untreated cells. A Kolmogorov-Smirnov test was used to test if the measurements were normally distributed or not. With very high confidence none of the speed and run length distributions was normal. The Wilcoxon rank sum test for equal medians of two continuous distributions was used to compare the median values of mEGFP-CC1 and p50-mEGFP distributions with the untreated cells. The null hypothesis was that the two independent samples had equal medians. The alternative hypothesis was that the samples did not have equal medians. Probability calculations of drawing a random sample of size s from the control condition without sampling values above the thresholds were performed (Figure 6c). The control dataset was significantly larger than the datasets of CC1-EGFP and p50-EGFP. We were concerned about the possibility that absence of segments with high speeds could be due to small datasets. Therefore, we estimated the likelihood of drawing a random sample of size s from the control dataset that had no values above a certain threshold T. In a first step the probability of experimental values below the threshold was calculated for the control dataset (P_low_ = events below threshold T/all events). The probability of drawing a random sample of size s with all values below P_low_ is:

$${\text{P}}_{\text{random}}={\text{P}}_{\text{low}}^{\text{s}}$$

The number of segments in the dataset of CC1-EGFP and p50-EGFP was used as s. The values for P_random_ were calculated. Values of P_random_ < 0.05 indicated a low probability of observing a distribution without values above the threshold T by chance.

## 5. Conclusions

The data presented here and earlier results show that dynactin supports microtubule-dependent directional motions of HAdV2/5 particles both towards and away from the nucleus, and thereby enhances the nuclear localization of HAdV2/5 and infection. How the viruses detach from microtubules near the nucleus, and attach the nuclear pore complexes for uncoating of their DNA is subject to future studies.

## Supplementary Materials

Supplemental Movie 1. Intracellular trajectories of HAdV2-atto565. HAdV2-atto565 was bound to HeLa cells at 4 °C for 30 min, unbound virus washed off, and cells were shifted to 37 °C for 47 min, followed by imaging of 2000 frames at an acquisition frequency of 25 Hz. Scale bar corresponds to 8 μm.

## Figures and Tables

**Table 1.** Dynactin interference reduces the number of directed motions (DM).

**Construct**	**No. of tracks**	**No. of DM runs**	**DM runs per track [%]**
mEGFP	1585	484	31%
mEGFP-CC1	1333	151	11%
p50-mEGFP	1829	354	19%

HeLa cells were transiently transfected with mEGFP, mEGFP-CC1 or p50-mEGFP and infected with HAdV2-atto565. Single particle tracking yielded cytoplasmic viral trajectories (Column 2), which were segmented into distinct motion patterns, such as directed motions (DM). Dynactin interference reduced the total number of DM (Column 3), and also the number of DM per track (Column 4).

## References

[1] Burckhardt CJ, Greber UF (2009). Virus movements on the plasma membrane support infection and transmission between cells. PLoS Pathog.

[2] Greber UF (2002). Signalling in viral entry. Cell. Mol. Life Sci.

[3] Bowie AG, Unterholzner L (2008). Viral evasion and subversion of pattern-recognition receptor signalling. Nat. Rev. Immunol.

[4] Mercer J, Schelhaas M, Helenius A (2010). Virus Entry by Endocytosis. Annu. Rev. Biochem.

[5] Harrison SC (2008). Viral membrane fusion. Nat. Struct. Mol. Biol.

[6] Mudhakir D, Harashima H (2009). Learning from the viral journey: How to enter cells and how to overcome intracellular barriers to reach the nucleus. AAPS J.

[7] Dohner K, Sodeik B (2005). The role of the cytoskeleton during viral infection. Curr. Top. Microbiol. Immunol.

[8] Greber UF, Way M (2006). A super highway to virus infection. Cell.

[9] Leopold PL, Pfister KK (2006). Viral strategies for intracellular trafficking: motors and microtubules. Traffic.

[10] Puntener D, Greber UF (2009). DNA-tumor virus entry—From plasma membrane to the nucleus. Semin. Cell Dev. Biol.

[11] Hsieh MJ, White PJ, Pouton CW (2010). Interaction of viruses with host cell molecular motors. Curr. Opin. Biotechnol.

[12] Vallee RB, Williams JC, Varma D, Barnhart LE (2004). Dynein: An ancient motor protein involved in multiple modes of transport. J. Neurobiol.

[13] Kardon JR, Vale RD (2009). Regulators of the cytoplasmic dynein motor. Nat. Rev. Mol. Cell Biol.

[14] Mallik R, Gross SP (2004). Molecular motors: Strategies to get along. Curr. Biol.

[15] Bornens M (2002). Centrosome composition and microtubule anchoring mechanisms. Curr. Opin. Cell Biol.

[16] Robinson CM, Singh G, Henquell C, Walsh MP, Peigue-Lafeuille H, Seto D, Jones MS, Dyer DW, Chodosh J (2011). Computational analysis and identification of an emergent human adenovirus pathogen implicated in a respiratory fatality. Virology.

[17] Hilleman MR, Werner JH (1954). Recovery of new agent from patients with acute respiratory illness. Proc. Soc. Exp. Biol. Med.

[18] Rowe WP, Huebner RJ, Gilmore LK, Parrott RH, Ward TG (1953). Isolation of a cytopathogenic agent from human adenoids undergoing spontaneous degeneration in tissue culture. Proc. Soc. Exp. Biol. Med.

[19] Berget SM, Moore C, Sharp PA (1977). Spliced segments at the 5′ terminus of adenovirus 2 late mRNA. Proc. Natl. Acad. Sci. U. S. A.

[20] Chow LT, Gelinas RE, Broker TR, Roberts RJ (1977). An amazing sequence arrangement at the 5′ ends of adenovirus 2 messenger RNA. Cell.

[21] Andersson M, Paabo S, Nilsson T, Peterson PA (1985). Impaired intracellular transport of class I MHC antigens as a possible means for adenoviruses to evade immune surveillance. Cell.

[22] Cepko CL, Sharp PA (1982). Assembly of adenovirus major capsid protein is mediated by a nonvirion protein. Cell.

[23] Stillman BW (1983). The replication of adenovirus DNA with purified proteins. Cell.

[24] Trentin JJ, Yabe Y, Taylor G (1962). The quest for human cancer viruses. Science.

[25] Doerfler W (2009). Epigenetic mechanisms in human adenovirus type 12 oncogenesis. Semin. Canc. Biol.

[26] Chailertvanitkul VA, Pouton CW (2010). Adenovirus: A blueprint for non-viral gene delivery. Curr. Opin. Biotechnol.

[27] Benson SD, Bamford JK, Bamford DH, Burnett RM (1999). Viral evolution revealed by bacteriophage PRD1 and human adenovirus coat protein structures. Cell.

[28] Reddy VS, Natchiar SK, Stewart PL, Nemerow GR (2010). Crystal structure of human adenovirus at 3.5 A resolution. Science.

[29] Liu H, Jin L, Koh SB, Atanasov I, Schein S, Wu L, Zhou ZH (2010). Atomic structure of human adenovirus by cryo-EM reveals interactions among protein networks. Science.

[30] Bergelson JM, Cunningham JA, Droguett G, Kurt-Jones EA, Krithivas A, Hong JS, Horwitz MS, Crowell RL, Finberg RW (1997). Isolation of a common receptor for Coxsackie B viruses and adenoviruses 2 and 5. Science.

[31] Tomko RP, Xu R, Philipson L (1997). HCAR and MCAR: The human and mouse cellular receptors for subgroup C adenoviruses and group B coxsackieviruses. Proc. Natl. Acad. Sci. U. S. A.

[32] Wickham TJ, Mathias P, Cheresh DA, Nemerow GR (1993). Integrins alpha v beta 3 and alpha v beta 5 promote adenovirus internalization but not virus attachment. Cell.

[33] Meier O, Boucke K, Hammer SV, Keller S, Stidwill RP, Hemmi S, Greber UF (2002). Adenovirus triggers macropinocytosis and endosomal leakage together with its clathrin-mediated uptake. J. Cell Biol.

[34] Gastaldelli M, Imelli N, Boucke K, Amstutz B, Meier O, Greber UF (2008). Infectious adenovirus type 2 transport through early but not late endosomes. Traffic.

[35] Greber UF, Willetts M, Webster P, Helenius A (1993). Stepwise dismantling of adenovirus 2 during entry into cells. Cell.

[36] Wiethoff CM, Wodrich H, Gerace L, Nemerow GR (2005). Adenovirus protein VI mediates membrane disruption following capsid disassembly. J. Virol.

[37] Nakano MY, Boucke K, Suomalainen M, Stidwill RP, Greber UF (2000). The first step of adenovirus type 2 disassembly occurs at the cell surface, independently of endocytosis and escape to the cytosol. J. Virol.

[38] Puntener D, Engelke M, Ruzsics Z, Strunze S, Wilhelm C, Greber U (2011). Stepwise loss of fluorescent core protein V from human adenovirus during entry into cells. J. Virol.

[39] Suomalainen M, Nakano MY, Boucke K, Keller S, Stidwill RP, Greber UF (1999). Microtubule-dependent minus and plus end-directed motilities are competing processes for nuclear targeting of adenovirus. J. Cell Biol.

[40] Leopold PL, Kreitzer G, Miyazawa N, Rempel S, Pfister KK, Rodriguez-Boulan E, Crystal RG (2000). Dynein- and microtubule-mediated translocation of adenovirus serotype 5 occurs after endosomal lysis. Hum. Gene Ther.

[41] Suomalainen M, Nakano MY, Boucke K, Keller S, Greber UF (2001). Adenovirus-activated PKA and p38/MAPK pathways boost microtubule-mediated nuclear targeting of virus. Embo. J.

[42] Mabit H, Nakano MY, Prank U, Saam B, Döhner K, Sodeik B, Greber UF (2002). Intact microtubules support adenovirus and herpes simplex virus infections. J. Virol.

[43] Kelkar SA, Pfister KK, Crystal RG, Leopold PL (2004). Cytoplasmic dynein mediates adenovirus binding to microtubules. J. Virol.

[44] Gazzola M, Burckhardt CJ, Bayati B, Engelke M, Greber UF, Koumoutsakos P (2009). A stochastic model for microtubule motors describes the in vivo cytoplasmic transport of human adenovirus. PLoS Comp. Biol.

[45] Bremner KH, Scherer J, Yi J, Vershinin M, Gross SP, Vallee RB (2009). Adenovirus transport via direct interaction of cytoplasmic dynein with the viral capsid hexon subunit. Cell Host Microbe.

[46] Kelkar S, De BP, Gao G, Wilson JM, Crystal RG, Leopold PL (2006). A common mechanism for cytoplasmic dynein-dependent microtubule binding shared among adeno-associated virus and adenovirus serotypes. J. Virol.

[47] Martinez-Moreno M, Navarro-Lerida I, Roncal F, Albar JP, Alonso C, Gavilanes F, Rodriguez-Crespo I (2003). Recognition of novel viral sequences that associate with the dynein light chain LC8 identified through a pepscan technique. FEBS Lett.

[48] Wodrich H, Henaff D, Jammart B, Segura-Morales C, Seelmeir S, Coux O, Ruzsics Z, Wiethoff CM, Kremer EJ (2010). A capsid-encoded PPxY-motif facilitates adenovirus entry. PLoS Pathog.

[49] Greber UF, Suomalainen M, Stidwill RP, Boucke K, Ebersold M, Helenius A (1997). The role of the nuclear pore complex in adenovirus DNA entry. EMBO. J.

[50] Trotman LC, Mosberger N, Fornerod M, Stidwill RP, Greber UF (2001). Import of adenovirus DNA involves the nuclear pore complex receptor CAN/Nup214 and histone H1. Nat. Cell. Biol.

[51] Wisnivesky JP, Leopold PL, Crystal RG (1999). Specific binding of the adenovirus capsid to the nuclear envelope. Hum. Gene Ther.

[52] Gill SR, Schroer TA, Szilak I, Steuer ER, Sheetz MP, Cleveland DW (1991). Dynactin, a conserved, ubiquitously expressed component of an activator of vesicle motility mediated by cytoplasmic dynein. J. Cell Biol.

[53] Schroer TA, Sheetz MP (1991). Two activators of microtubule-based vesicle transport. J. Cell Biol.

[54] Karki S, Holzbaur EL (1999). Cytoplasmic dynein and dynactin in cell division and intracellular transport. Curr. Opin. Cell Biol.

[55] King SJ, Schroer TA (2000). Dynactin increases the processivity of the cytoplasmic dynein motor. Nat. Cell Biol.

[56] Schroer TA (2004). Dynactin. Annu. Rev. Cell Dev. Biol.

[57] Quintyne NJ, Gill SR, Eckley DM, Crego CL, Compton DA, Schroer TA (1999). Dynactin is required for microtubule anchoring at centrosomes. J. Cell Biol.

[58] Imelli N, Ruzsics Z, Puntener D, Gastaldelli M, Greber UF (2009). Genetic reconstitution of the human adenovirus type 2 temperature-sensitive 1 mutant defective in endosomal escape. Virol. J.

[59] Sbalzarini IF, Koumoutsakos P (2005). Feature point tracking and trajectory analysis for video imaging in cell biology. J. Struct. Biol.

[60] Helmuth JA, Burckhardt CJ, Koumoutsakos P, Greber UF, Sbalzarini IF (2007). A novel supervised trajectory segmentation algorithm identifies distinct types of human adenovirus motion in host cells. J. Struct. Biol.

[61] Burkhardt JK, Echeverri CJ, Nilsson T, Vallee RB (1997). Overexpression of the dynamitin (p50) subunit of the dynactin complex disrupts dynein-dependent maintenance of membrane organelle distribution. J. Cell Biol.

[62] Zacharias DA, Violin JD, Newton AC, Tsien RY (2002). Partitioning of lipid-modified monomeric GFPs into membrane microdomains of live cells. Science.

[63] Welte MA (2004). Bidirectional transport along microtubules. Curr. Biol.

[64] Gross SP, Vershinin M, Shubeita GT (2007). Cargo transport: two motors are sometimes better than one. Curr. Biol.

[65] Muller MJ, Klumpp S, Lipowsky R (2008). Tug-of-war as a cooperative mechanism for bidirectional cargo transport by molecular motors. Proc. Natl. Acad. Sci. U. S. A.

[66] Yue L, Lu S, Garces J, Jin T, Li J (2000). Protein kinase C-regulated dynamitin-macrophage-enriched myristoylated alanine-rice C kinase substrate interaction is involved in macrophage cell spreading. J. Biol. Chem.

[67] Hoogenraad CC, Akhmanova A, Howell SA, Dortland BR, De Zeeuw CI, Willemsen R, Visser P, Grosveld F, Galjart N (2001). Mammalian Golgi-associated Bicaudal-D2 functions in the dynein-dynactin pathway by interacting with these complexes. Embo. J.

[68] Haghnia M, Cavalli V, Shah SB, Schimmelpfeng K, Brusch R, Yang G, Herrera C, Pilling A, Goldstein LS (2007). Dynactin is required for coordinated bidirectional motility, but not for dynein membrane attachment. Mol. Biol. Cell.

[69] Ally S, Larson AG, Barlan K, Rice SE, Gelfand VI (2009). Opposite-polarity motors activate one another to trigger cargo transport in live cells. J. Cell Biol.

[70] Deacon SW, Serpinskaya AS, Vaughan PS, Lopez Fanarraga M, Vernos I, Vaughan KT, Gelfand VI (2003). Dynactin is required for bidirectional organelle transport. J. Cell Biol.

[71] Culver-Hanlon TL, Lex SA, Stephens AD, Quintyne NJ, King SJ (2006). A microtubule-binding domain in dynactin increases dynein processivity by skating along microtubules. Nat. Cell Biol.

[72] Kardon JR, Reck-Peterson SL, Vale RD (2009). Regulation of the processivity and intracellular localization of Saccharomyces cerevisiae dynein by dynactin. Proc. Natl. Acad. Sci. U. S. A.

[73] Berezuk MA, Schroer TA (2007). Dynactin enhances the processivity of kinesin-2. Traffic.

[74] Snapp EL, Hegde RS, Francolini M, Lombardo F, Colombo S, Pedrazzini E, Borgese N, Lippincott-Schwartz J (2003). Formation of stacked ER cisternae by low affinity protein interactions. J. Cell Biol.

[75] Kreis TE (1987). Microtubules containing detyrosinated tubulin are less dynamic. Embo. J.

